# Baltimore community resident and collaborator perspectives on the influence of guaranteed income on health: a formative qualitative study

**DOI:** 10.1186/s12889-024-19771-5

**Published:** 2024-09-18

**Authors:** Holly M. Nishimura, Sevly Snguon, Lorraine T. Dean, Marik Moen

**Affiliations:** 1 Division of Prevention Science, University of California School of Medicine UCSF, 550 16th Street, 3rd Floor, Box 0886, 94143 San Francisco, CA USA; 2 Department of Epidemiology, Johns Hopkins Bloomberg School of Public Health, Baltimore, MD USA; 3 Family and Community Health, University of Maryland School of Nursing, Baltimore, MD USA

**Keywords:** Guaranteed income, Baltimore, Health

## Abstract

**Background:**

Given increased interest and investment in guaranteed income initiatives across North America, it is critical to understand the impact of guaranteed income on health, an understudied outcome. As part of Baltimore, Maryland’s guaranteed income pilot, we conducted formative research to understand the influence of increased income on health and develop concrete recommendations for implementation and evaluation of the pilot.

**Methods:**

We conducted semi-structured in-depth interviews with Baltimore community residents (*n* = 8) and community collaborators (*n* = 8), probing on familiarity with guaranteed income; effect of guaranteed income on overall health and specific health outcomes (e.g., mental health, nutrition); and recommendations for program structure (e.g., eligibility, target population). Data were analyzed thematically using a framework analysis approach.

**Results:**

Respondents agreed that guaranteed income could have beneficial effects on health though some were unsure of specific mechanisms. Respondents emphasized pathways through which guaranteed income could improve health: (1) reduced financial-related stress; (2) improved nutrition through purchase of healthier foods; (3) improved family well-being including child health and parent-child relationships; (4) increased utilization of health services; (5) improved community health through increased community cohesion and decreased violence. Respondents described decreased feelings of time scarcity as a social determinant of health. Most respondents reported that Baltimore’s guaranteed income program should prioritize young, low-income families with $1,000/month or more for at least one year.

**Conclusions:**

This formative research on the potential health impacts of guaranteed income in Baltimore highlights important health outcomes and pathways, such as social cohesion and decreased feelings of time scarcity, to prioritize for evaluation.

**Supplementary Information:**

The online version contains supplementary material available at 10.1186/s12889-024-19771-5.

## Introduction

**Fig. 1:**
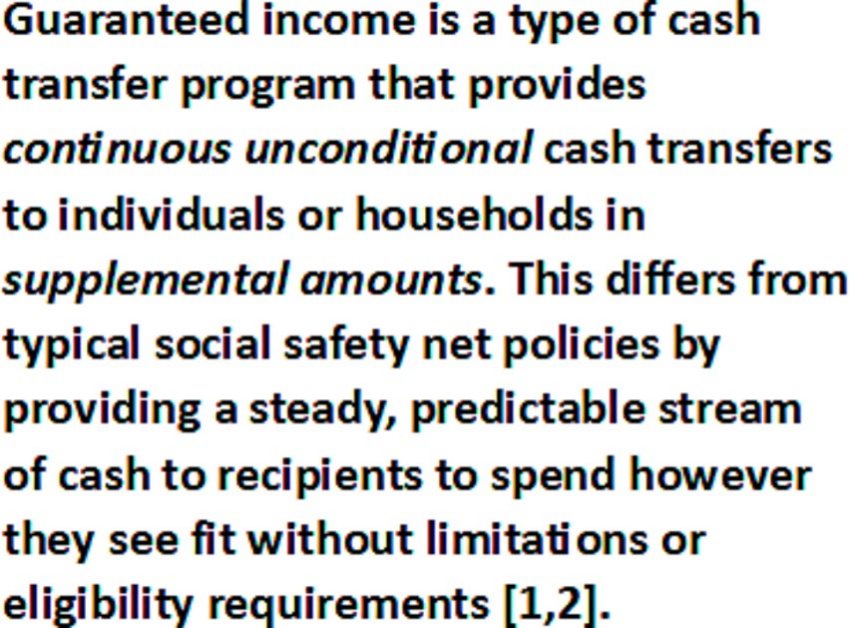
Definition of guaranteed income

The United States has seen a precipitous increase in guaranteed income (GI) in recent years. GI provides an income floor and supplements social safety net programs, whereas other cash transfer initiatives, such as universal basic income, aim to provide enough income to meet basic needs of members of the community (defined in Fig. 1) [1, 2].

From its introduction in the US in the 1960’s, guaranteed income has been conceived as a policy approach to reduce income inequality and improve access to critical resources such as stable housing and education for low-income individuals and families [3]. These recurring, no-strings attached direct cash transfer income supplements provided to individuals and households over time have become part of mainstream national and regional policy discourse in the U.S [4, 5]. In 2022 over 100 mayors had adopted guaranteed income programs in 31 cities across the U.S. as part of the Mayor’s for a Guarantee Income Initiative (MGI) [6], in addition to other demonstration projects across the country.

Income is associated with health through a direct effect on the material conditions necessary for biological survival, and through an effect on social participation and opportunity to control life circumstances [7, 8]. While there have been numerous studies on the health impacts of conditional and unconditional cash transfers on health in lower- and middle-income countries [3, 9, 10] fewer have examined the health impact and mechanisms of the growing number of recurring, unconditional, unrestricted, supplemental (guaranteed income) cash transfers. Prior guaranteed income research hints at possible direct and indirect effects on health; however, findings have been inconsistent and there is little focus on research in high-income settings, and the mechanisms through which guaranteed income influences health are not well understood [3, 10–14]. High-income contexts and different forms of cash transfers may have varied influences on health [12].

Due to multi-level and complex intervening factors, linking social policy interventions to health outcomes is a complicated endeavor and substantial evidence for the effect of guaranteed income initiatives on health still is lacking. Even when clinical trials or epidemiologic studies demonstrate significant quantitative relationships of sufficient magnitude between interventions and outcome [10–12, 15, 16], they often lack information on the underlying mechanisms of why these relationships exist and how these relationships play out in people’s lives which are best explored through qualitative research.

Given the increased interest and investment in guaranteed income initiatives, it is critical to understand how guaranteed income may impact health and to explore potential mechanisms. As part of the Mayor’s for Guaranteed Income (MGI) Initiative in Baltimore, Maryland, the first health-focused MGI demonstration in the country, we conducted formative qualitative study to answer the research question, “How do Baltimore City low-income community residents and community collaborators think guaranteed income will influence recipients’ health outcomes, and what implementation strategies could maximize potential benefits to health?”

## Methods

### Study design

We conducted a cross-sectional qualitative study involving semi-structured in-depth interviews (IDIs) with 16 Baltimore City community residents (*n* = 8) and community collaborators (*n* = 8). We applied a qualitative description (QD) methodology as QD recognizes the shared and inseparable nature of humans from their experiences. We strived to describe individual experience in participants’ own words, and via analysis arrive at common themes, to develop a representation of the data of many individuals into common collective ideas [17].

### Study setting

This study was conducted in Baltimore, Maryland in July-December 2021 prior to the 2022 launch of Baltimore’s guaranteed income pilot. Baltimore has a population of 576, 498 [18] and is the third largest city in the mid-Atlantic region. 62% of the population identify as Black or African American while approximately 30% identify as white [18]. In the year prior to the study (2020), the median household income was [18] $52,164, and 20% of the population lived at or under the federal poverty line [18].

### Recruitment and sampling

We developed a pragmatic recruitment and sampling strategy with modest sample size to inform Baltimore MGI prior to implementation. Guidance on qualitative studies suggest 12 to 30 interviews may be needed to reach saturation [20]. We recruited and purposively sampled 16 individuals from two subgroups: (1) community residents (*n* = 8) and (2) community collaborators (*n* = 8). Community residents were current Baltimore City residents, ages 18 and above, who may be eligible to participate in cash transfer initiatives. We use the term community collaborators to designate key informants from partner organizations- Baltimore city employees, members of the Baltimore Mayor’s for Guaranteed Income project community advisory board, or staff from community-based organizations- who implemented cash transfer initiatives in Baltimore City. All individuals who were recruited agreed to participate in the study. Community collaborators were identified by the MGI working group as having a wealth of experience providing services to low-income Baltimore city residents and/or implementation of cash transfer programs. Community residents were recruited by community collaborators who referred clients who may be eligible for cash transfer initiatives based on their income or who had previously participated in a COVID-19 cash transfer program for Baltimore families.

### Data collection

After obtaining oral consent to participate in the study, all IDIs were conducted remotely over Zoom to protect participants and study staff from potential COVID-19 exposure. IDIs were audio recorded and transcribed verbatim using Zoom software [21]. Two researchers compared each transcript against the transcription to check for accuracy and made corrections as necessary. Using a semi-structured interview guide based on a cash transfer and health framework [22], IDIs probed on familiarity with guaranteed income initiatives; recommendations for optimal guaranteed income initiative structure (eligibility or target population, amount, duration); the effect of guaranteed income on overall health, maternal and child health, cardiovascular health, infectious disease, diet/nutrition, physical activity, mental health, substance use, violence, healthcare access or utilization, and potential community-level impacts on health. At the end of the interview, participants were asked to provide their age, occupation, race/ethnicity, gender, and income (community residents only). Following the interview, study staff de-identified and independently reconciled transcripts with audio recordings. Each participant was offered a $50 gift card for their participation.

### Data analysis

Following close reading of several transcripts, one member of the study team (HN) conducted line-by-line manual coding to become familiar with the data and identified potential emerging themes. We then used a framework analysis approach [22] a versatile qualitative method often used in health research, to analyze qualitative data. First, team members abstracted qualitative data into a framework table in Microsoft Excel, where rows are participants’ data, and the columns are pre-defined categories for theme organization based on interview guides (available in supplemental materials). Framework table contents were systematically assessed for patterns, further developed, analyzed, and refined. Multiple reviewers contributed to developing emerging themes, and in next level analyses developed final themes with illustrative quotes on a second Excel table. Framework analysis allows for multidisciplinary team members to collaborate in a highly structured, providing transparency as the framework evolves. This approach is aligned with qualitative description designs and facilitates thematic analysis for the purposes of informing policy development, as in the case of this project [22].

### Reflexivity statement

All authors are employed at public health academic institutions. Prior to their academic work, one author (SS) worked for an advocacy organization focused on anti-poverty initiatives including guaranteed income. All authors come from family backgrounds where family members would likely be eligible for guaranteed income. One author (SS) participated in a community collaborator interview. Results must be considered in light of an authorship team that is invested in making links to social factors and health.

### Ethical considerations

This study was approved by the Johns Hopkins Bloomberg School of Public Health IRB (protocol #16992) exempt with no or minimal risk to human subjects.

## Results

### Sample characteristics

Table 1 shows the demographic characteristics of the sample. All community resident respondents (*N* = 8) identified as Black or African American and ranged in age from 27 to 58. All community residents received secondary-level education and three had attended college. All community residents earned less than Baltimore’s median income ($52,164 in 2020) and five residents earned less than the federal poverty level for a household of three ($21,720 in 2020). Four community residents had previously participated in a cash transfer program and none of the community residents had ever heard of guaranteed income prior to the interview.

Community collaborator respondents were employed with the City of Baltimore, nonprofits, or medical centers serving communities who could be eligible for Baltimore’s guaranteed income initiative. Five community collaborators identified as white, three identified as Black or African American, and one identified as Asian American; they ranged in age from 32 to 58. All community collaborators received graduate-level education. Five community collaborators had direct experience with cash transfer initiatives including serving on the Mayor’s for Guaranteed Income working group or implementing previous cash transfer initiatives.

**Table 1:** Interviewee characteristics

	Community Residents (*n* = 8)	Community Collaborators (*n* = 8)
**Age**: mean (standard deviation)	40 (11)	43 (8)
**Race**		
Black or African-American	8	3
White	0	4
Other	0	1
**Gender**		
Female	4	7
Male	4	0
Not reported	0	1
**Education**		
High school	3	0
Some college/bachelor’s degree	2	0
Graduate degree	0	8
**Annual income**		
*≤*$20,000/year	5	N/A
>$20,000/year	3	N/A
**Cash transfer participation**	4	N/A
**Knowledge of** **guaranteed income**	0	5

*Note* Community collaborators were not asked income and cash transfer participation and knowledge questions

### Key findings

Community residents and community collaborators explained whether and how guaranteed income could affect the health of individuals, families, and communities in Baltimore. Below, we summarize their responses by health outcome. Respondents also provided their perspectives on the structure and implementation of Baltimore’s Guaranteed Income Initiative.

### Health outcomes and mechanisms

Most community residents and community collaborators had not thought about the connection between income and health prior to the interview. When asked if increased income through a guaranteed income initiative would influence their health, the preponderance of respondents thought that guaranteed income could improve health; however, four respondents thought there would be no effect or were unsure of the pathways through which guaranteed income could impact health.

*I mean*,* I could really only conjecture. I am not sure I know that I could answer that*,* aside from looking at how these initiatives have already… I just don’t know. IDI 11*, *community collaborator*.

### Mental health, stress, and family wellbeing

All community residents and collaborators strongly emphasized the strong connection between financial instability and poor mental health, namely stress, and how that might be reflected by changes in physical health. For example, one community resident described how stress related to financial strain could directly impact her own physiological health through changes in levels of cortisol, a stress hormone, which could have secondary impacts of poor cardiovascular health.

*I think that we don’t realize the huge effect of stress and even with just cortisol hormones*,* I might start gaining more weight around my midsection which makes me more vulnerable to have a heart attack. IDI 12*,* community resident*.

Respondents described the burden of financial stress related to family health, particularly for mothers preoccupied with caring for their children. One respondent explained the difficulty of prioritizing her own health while also caring for her children. The challenges were compounded by other financial-related stressors which could ultimately impact physiological health.

*Especially moms*,* we’re so focused on our children and their health*,* we forget to take care of ourselves so adding on that extra layer of stress of job insecurity or housing insecurity or things like that that could be detrimental to health. So I think the guaranteed income could definitely have a positive impact when we look at other health outcomes like cardiovascular disease or obesity or things like that. IDI 3*,* community resident*.

Community residents and collaborators alike described how by relieving financial-related stressors for individual recipients, guaranteed income would indirectly impact relationships with their children and improve family dynamics.

*When every time you ask for something*,* [it’s] ‘we don’t have money for that*,* or that’s not available’…it [ guaranteed income] could help*,* you know*,* to even decrease the parents’ anxiety and then*,* in turn*,* the child’s anxiety. IDI 12*,* community resident*.

Lastly, respondents described how increased income could address multiple dimensions of stress associated with financial strain. For example, a number of community residents described how additional income could be used to treat themselves to small luxuries that would alleviate stress.

A strong theme among respondents was agreement that additional income would have a positive impact on mental health. Others felt that guaranteed income alone could not address existing barriers to mental health services, such as stigma, that would be needed to improve mental health outcomes. One community collaborator explained:

*I mean there’s a lot that has to be done around mental health and trauma that this money is not going to address it’s you know sure I guess you could say then maybe some will go to a therapist or something like that*,* but there’s too much stigma around going to therapy still. IDI 7*,* community collaborator*.

### Substance use

Similar to stressors that affect mental health and family wellbeing, a major theme among community residents was that substance use was a result of stressful economic situations, especially not being able to afford basic things or needing to engage in substance use as a coping mechanism. Several community residents reported that additional income could reduce the need for temptation goods such as alcohol, cigarettes, or illicit substances.

*I don’t think that’ll increase it [substance use]*,* if anything*,* it may help to decrease it…There would (be) more hope for the future. There is less need for escapism… And I think if there was like some kind of hope*,* something to look forward to*,* and something that you could see as a stepping stone that would discourage the [substance] use because ‘this could be my chance*,*’ like you know what I mean? I feel like they could think in their head like*,* ‘This could be my chance by get myself together get myself on the right track.’ IDI 12*,* community resident*.

While community residents consistently reported the potential positive effects of additional income on substance use, one community resident and two community collaborators expressed concern that funds could be used to purchase illicit substances. One community collaborator explained that additional income might increase substance use but could also be associated with the safer use of substances or prevent recipients from “doing something desperate to obtain substances.”- *IDI 2*,* community collaborator*.

### Nutrition

The link between financial insecurity and food insecurity, --with poor nutrition or improved nutrition contributing to health outcomes– was another major theme. Community residents and collaborators agreed that guaranteed income could buy higher-quality, nutritious foods rather than lower-cost options which tended to be unhealthy, with benefits be particularly important for pregnant women.

*[Right now]*,* I gotta get poor food*,* so you get salty food*,* get the lowest of the lowest soda that’s cheap so that’s why people have diabetes problems*,* stuff like that. You get a lot of bread*,* so you can fill your stomach up. IDI 15*,* community resident*.

*Also [women] having a child - I learned eating fish*,* certain fruits*,* and vegetables are healthy for helping the baby grow in you so that would definitely help give a woman better choices of meals to eat*,* while she’s carrying a child. IDI 10*,* community resident*.

### Healthcare utilization and access

Community collaborators emphasized how guaranteed income could improve access to and utilization of healthcare services. One community collaborator suggested that guaranteed income could be a gateway to health and social services which would further expand recipients’ access to other services. Two community collaborators thought that the guaranteed income initiative could be an important way to increase utilization of dental care which is not covered by public insurance policies, such as Medicare.

*I think I’ve heard somewhere that you know healthcare is one of those sectors or services where*,* once you start becoming a more regular user of health services*,* you get more exposure to other available health services and so you end up kind of it’s called like an inexhaustible good but once you have access*,* you start kind of having more and more access. IDI 1*,* community collaborator*.

*I also think about like I’m like health integration too primarily because people will have more resources. …Where people want to seek more support*,* more resources*,* more mental health*,* more health care right they might see that as accessible now. Especially for particular things*,* like dental care and a lot of these are not covered. IDI 5*,* community collaborator.*

Community residents did not describe a connection between guaranteed income and healthcare access and utilization as often as community collaborators. However, one community resident emphasized how guaranteed income could increase the ability to access preventive health/holistic wellness services she would not otherwise have access to.

*It would 100% affect my health*,* because I believe in like holistic measures*,* whether it’s acupuncture or reiki and things like that… I would definitely use it [extra income] for things to help me to*,* you know*,* to thrive…so I’m thinking more of a wellness plan if people are using it for things like that I feel like it would help mental [and] physical health. IDI 13*,* community resident*.

### Community health

Community residents thought that a guaranteed income initiative would have an impact on interpersonal relationships and the health of communities, even if only some of the community residents were guaranteed income recipients. Both community resident and community collaborator respondents explained that improved financial stability could directly reduce stress and feelings of scarcity which could lead to an improved positive outlook and greater focus on the needs of others.

*Within the community*,* now you know*,* there is no loyalty amongst communities now. They could see my child out front hungry and walk right by ‘em [but when] I have a little to spare…and I see that the kid next door is struggling a little bit*,* the money I do receive*,* I maybe can buy a sandwich today or get him ice cream off the ice cream truck today*,* you know*,* so I think a little money in a home can improve [the community]. IDI 8*,* community resident*.

One community collaborator described how recipients would directly benefit from guaranteed income and could then be empowered to reinvest in their communities.

*The people who don’t have the means to either support their families or themselves experience a lot of shame and they can withdraw as a result of that and self-isolate and not engage. I think when you can look to your neighbor and everyone has been lifted*,* some of that shame goes away. Even though it is a subsidy*,* I think it’s something that can have a huge impact on an entire community…on the giving level*,* there are many*,* many people that would reinvest that funding into the community. IDI 7*,* community collaborator*.

Another community collaborator described that in marginalized communities, scarcity can lead to violence and crime, which might be reduced by an infusion of resources. They further explained that additional income could generate a greater sense of social cohesion, or solidarity, among people in the community which could lead to reductions in violence.

*[C]ommunities who come from poverty*,* um there’s a lot of historical violence also inter- community violence too like people will take from each other*,* because there isn’t an abundance of resources*,* because they were positioned in neighborhoods of poverty*,* so I think that*,* like social cohesion can improve because there’s more trust and people have what they need right where they feel that they might not need to take away from each other…we might see like reductions and petty crime or whatever. IDI 5*,* community collaborator*.

Other respondents, both community residents and community collaborators, did not think that guaranteed income would have health effects at the community level, including crime and violence. While some respondents explained that community violence could be reduced with increased income but were unsure of whether it would have a meaningful impact.

*I don’t know how the cash would help assist with violence; I don’t know if it would…We all know the research that shows that there will be a decrease in violence because of an increase in cash when clearly there would be*,* right*,* people would have jobs and people would be able to move out of poverty if they had what they would need to be able to be supported*,* but not exactly sure how*,* how big of a jump that would be. IDI 4*,* community collaborator*.

### Social determinants of health

A major theme among respondents was the potential for guaranteed income to influence social determinants of health beyond the direct effect on financial stability and poverty alleviation. Respondents also explained that consistent access to additional funds would afford recipients access to better neighborhoods, housing, and housing repairs that could reduce the risk of harmful exposure to lead or environmental pollutants.

*Think about all the projects*,* even in my own house that*,* sometimes the barriers is time*,* but in some households*,* the barrier is money…There was a family that I knew*,* who had a rodent infestation and just didn’t know how to get that under control without spending a lot of money and that was a real expense that was not planned for… certainly would have impacted that family’s health over time if it hadn’t been addressed. IDI 1*,* community collaborator*.

Many respondents reported that the influx of income would allow program participants to reclaim their time and mental bandwidth to engage in healthier behaviors such as increased physical activity.

.*having time to exercise and get out with kids or go for a walk or just be outside - time that isn’t spent in another job that would be very helpful - you know*,* being able to take advantage of some other hours in the day besides putting on the next uniform and heading out to work again. IDI 7*,* community collaborator*.

*And it’s still hugely frustrating and very time consuming and*,* like the time that I could be using try and find a job I’m stressed out like and I’m trying to figure out okay well how do I navigate this. IDI 12*,* community resident*.

Further, respondents emphasized that increased income could expand choices and opportunities related to healthcare needs, transportation, and especially employment. One community resident with experience applying for unemployment benefits described how guaranteed income could free up the significant time and energy costs associated with applying for services and benefits.

*I think like*,* even the choices that maybe that extra money gives someone the opportunity to have a different form of employment that may be less stressful or…They can pay for daycare so they can have a job that is more fulfilling*,* or they increase their earning power. IDI 12*,* community resident*.

### Recommendations for guaranteed income initiative implementation and structure

In addition to providing their thoughts on the potential effects of guaranteed income on health, respondents offered their recommendations on the implementation of Baltimore’s guaranteed income initiative including program eligibility, target population, disbursement amount, and program duration. Recommendations are summarized in Table 2.

Respondents recommended income-based eligibility for participation in guaranteed income initiatives. For example, one community collaborator suggested eligibility based on neighborhood-level income focusing on historically underserved areas of Baltimore rather than household-income. Several respondents stated that that youth, low-income families, and single mothers would benefit most from additional income and should be prioritized as recipients for Baltimore’s guaranteed income initiative.

Respondents recommended a range of $300-$2,000 as the monthly distribution amounts for the guaranteed income initiative. Almost all (*n* = 13) respondents expressed that at least $1,000 should be distributed to be effective while others suggested lesser amounts to cover a subset of specific basic needs. A duration of at least one year was recommended by all respondents. Three respondents felt that a minimum of two years was required for the initiative to make a positive difference in health outcomes.

Community residents and collaborators expressed concerns related to eligibility, program structure, and how funds would be used. One community collaborator was concerned about whether additional income would preclude families from eligibility for other income-based services. A small number of community residents and community collaborators voiced concerns about the unconditionality component of the guaranteed income initiative, specifically the misuse of funds for illicit substances or the purchase of “unnecessary” items.

**Table 2:** Summary of recommendations for Baltimore’s guaranteed income pilot

Program eligibility	Based on income, dependents, and/or household expenses
**Target population**	Low-income families, particularly single mothers
**Distribution amount**	Recommended *≥*$1000/month (range: $300–2000/month)
**Program duration**	*≥* 1 year
**Additional considerations**	Concern over money spent on “unnecessary” or luxury items or illegal substances

## Discussion

This research expands upon existing literature examining the impact of cash transfers on health. This qualitative study conducted with community residents and community collaborators will inform the implementation and evaluation of a guaranteed income initiative and help to understand the potential impacts and mechanisms of guaranteed income on health outcomes.

### Guaranteed income and financial strain

We focus on guaranteed income in our study, as distinct from universal basic income (UBI), two policy approaches to address poverty. The goal of UBI is to provide enough income to meet basic needs of recipients. Guaranteed income provides an income floor that elevates our most vulnerable populations and supplements social safety net programs. This distinction is important in the U.S. context. The Department of Health and Human Services issues poverty guidelines that serve as income eligibility levels for social safety net programs. As Baltimore’s Young Family Success Fund (BYFSF) was starting, $21,720 was the Federal Poverty Guideline for a family of three. Meanwhile, a living annual income, or the required amount for meeting basic needs of living, for a family of three in Baltimore is estimated at $131,019 [23]. Applicants to the BYFSF project reported median annual incomes of $7,350 and median average incomes of around $15,071. By either metric, even an influx of up to $12,000 per year to recipient households via a guaranteed income program is not sufficient to raise incomes to living wage standards in Baltimore, though it may still help them. Indeed, our study respondents universally agreed that guaranteed income would have an immediate and direct effect on relieving financial strain, particularly among parents. Our findings align with those of the Stockton Economic Empowerment Demonstration (SEED) [24], which showed that guaranteed income can have significant improvement in emotional health among recipients compared to baseline measurements, likely due to the lifting of some emotional strain due to financial strain. *(See Table in Supplemental Materials for characteristics of guaranteed income initiatives described in discussion.)*

#### Guaranteed income and family health

Improved nutrition was the most emphasized theme by respondents in our study as a direct and immediate outcome of guaranteed income. A respondent mentioned how additional income could improve maternal nutrition. SEED’s preliminary evaluation found that recipients spent the largest proportion of the cash transfer on food for the household which was inadequately covered by food stamp benefits [24]. Both the Alaska Permanent Fund and the Healthy Baby Prenatal Benefit reported improvements in birth outcomes among recipients which they attributed to improved maternal nutrition and overall health [25, 27]. These findings with guaranteed income recipients mirror behaviors of people in the US across income bracket. Food accounts for the greatest share of income spending in lowest income quintiles, and spending on food increases as incomes rise, even as the share of spending on food becomes smaller at higher income levels [28].

Respondents also emphasized how guaranteed income could improve family well-being through various mechanisms. Increased income could reduce financial strain and afford parents more quality time to spend with their children. This finding is somewhat aligned with a finding from the Alaska Permanent Fund which reported improvements in consistent parenting but these improvements were restricted to mothers with low-education [24]. Similarly, improvements in parent-child relationship quality and parental supervision were reported by recipients of tribal dividends [25].

Another theme that evolved in interviews was how guaranteed income could decrease feelings of time scarcity or contribute to feeling less strapped for time. Having more time was viewed as an important factor contributing to health outcomes including mental health and parent-child relationships. Qualitative data from SEED recipients supports the idea of time as a determinant of health. SEED recipients recalled how increased income allowed to them to participate in activities that were previously inaccessible due to financial constraints, spend meaningful time with their children, and do “normal things that a lot of people take for granted” [24]. Time has been identified as a social determinant of health [29](Gee et al., 2019)] that becomes scarcer when people with low resources and who are affected by racism must take more time to access support for daily needs (e.g. longer commute times to work due to spatial mismatch; being made to wait in long lines to access services; facing life disruption due to social inequities like unjust criminalization). Our results suggest that guaranteed income would enable people to reclaim more time for family and other needs.

### Indirect and long-term impacts

Mental health, nutrition, and birth outcomes are commonly evaluated outcomes of cash transfers [14, 25, 26]. The indirect and long-term impacts of cash transfers on health are still not well understood. Our findings related to the spill-over or indirect effects leading to improvements in community health through increased social cohesion and community engagement and reinvestment have not been examined thoroughly in cash transfer studies. The one-year evaluation of SEED did not observe any changes in the amount of funds spent on donations; however, community reinvestment may be in the form of time rather than donation amounts [24]. Our study respondents articulated that substance use and crime were related to feelings of financial strain, frustration, and desperation and these feelings influence individuals and the health of the community environment. Baltimore has high levels of crime and homelessness for a city of its size, that may contribute to reduced community quality of life [30, 31]. Guaranteed income may influence reduction in violence and crime, as observed among recipients of the Alaska Permanent Fund [32], Rural Income Maintenance Experiment [33], and casino dividends [26], which could impact community health.

The effect of guaranteed income initiatives on safety, behavioral health, and community quality of life, or community-level spill-over effects, may take more time to develop than other health outcomes and therefore would not be captured in a one-year evaluation. This delayed effect may be especially true in Baltimore where frustrations related to financial strain may be acutely experienced in some, predominantly Black, historically redlined communities where 31–50% of households live below the federal poverty level, especially given the gap between current income levels and what is needed for a living wage [23, 34].

#### Recommendations for implementation and evaluation

This study reveals findings that can be used to improve implementation and messaging related to guaranteed income and improve public perception of these initiatives. Unconditional cash transfer and guaranteed income initiatives often suffer from public misconceptions that low-income people are less responsible with their money than those in other income brackets [32–33, 35–36 ]. Guaranteed income initiatives can emphasize that recipients use the majority of funds to meet basic needs (e.g., food, utilities, transportation) [24] and that the purchase of items that may be considered “unnecessary” can play an important role in relieving stress as one respondent mentioned.

Some respondents expressed concern over the use of funds for temptation goods such as illicit substances, alcohol, and tobacco. There is inconsistent evidence on the effect of increased income on purchase of temptation goods with both small increases or decreases documented [24, 32, 35–37]. Recent data from SEED show that less than 1% of tracked purchases were used for tobacco and alcohol [24]. Given these inconsistent findings, guaranteed income initiatives should evaluate substance use patterns over time and consider ways to enhance linkage of recipients to social and health services, such as substance use treatment programs.

Our findings can inform the methods and timing for evaluation of the effects of guaranteed income on health. Evaluations of cash transfers have focused primarily on the immediate and direct effects on health (e.g., stress levels, purchase of healthier foods), and healthcare utilization (e.g., hospitalizations). Our study suggests important indirect and spill-over health effects and health effects that occur with extended periods of increased income, for example 1–5-year evaluations of maternal and child health, environmental exposures, and body mass index or longer-term (> 5 years) evaluations of cardiovascular disease and community social cohesion and violence. Further, qualitative analyses and mediation analyses are needed to explore mechanisms of effects on health and the extent to which mediating factors (e.g., psychological well-being, housing, education, time scarcity) impact health outcomes.

Our results can also be used to guide the development of frameworks that outline relationships to be explored in future analyses and implementation of guaranteed income projects. Publishing a framework is beyond the scope of this paper alone as it is part of forthcoming mixed methods work [38. Our results suggest that a framework should incorporate elements of short and long-term health effects, and social determinants of health constructs, to illustrate relationships of guaranteed income projects and health.

#### Strengths and limitations

A major strength of this research is the opportunity to inform the implementation and evaluation of Baltimore’s guaranteed income pilot. Our findings are rooted in the voices and perspectives of Baltimore community residents which is another strength of this study. This study used a small sample that may lack sufficient representation for generalizability of findings. Given the relatively narrow scope of Baltimore City community collaborators who have implemented unconditional cash transfers or worked extensively with low-income Baltimore City residents and Baltimore City residents who have received unconditional cash transfers or would be eligible for Baltimore’s guaranteed income demonstration, our study offers depth and richness of data appropriate for the research question. Given the qualitative nature of the study, our findings are not representative of all Baltimore communities or individual perspectives, however, our sampling strategy was devised to capture those with the most in-depth knowledge on the communities who would benefit from guaranteed income. Several community residents had no prior knowledge of or experience with cash transfers, including guaranteed income, and therefore the interview was the first time they considered potential health impacts. A majority of community collaborators had prior experience implementing cash transfers or worked directly with Baltimore’s guaranteed income initiative and offered more detailed information on the impact on health and potential mechanisms. We have attempted, in our write-up of these findings, to represent community resident and community collaborator perspectives with equal weight.

## Conclusion

This formative research with community residents and collaborators on the potential health impacts of guaranteed income in Baltimore highlights important health outcomes and pathways, such as social cohesion and decreased feelings of time scarcity, which should be prioritized for future evaluation of similar projects.

## Electronic supplementary material

Below is the link to the electronic supplementary material.

Supplementary Material 1

Supplementary Material 2

Supplementary Material 3

## Data Availability

All qualitative data are deidentified, available from the corresponding author on reasonable request with data use agreement.
