# Supplementary material for: Baltimore community resident and collaborator perspectives on the influence of guaranteed income on health: a formative qualitative study
Source: BMC Public Health. 2024 Sep 18;24:2537. doi: 10.1186/s12889-024-19771-5 (PMC11409528; doi:10.1186/s12889-024-19771-5)
Supplement: Supplementary file 1 — Supplementary Material 1 [file 12889_2024_19771_MOESM1_ESM.docx]

**Supplement**

**Table. Characteristics of guaranteed income initiatives described in discussion**

| **Name** | **Description** |
| --- | --- |
| Stockton Economic Empowerment Demonstration (SEED)^1^ | SEED was the first mayor-led guaranteed income demonstration in the U.S. The program provided 125 adults residing in low-income neighborhoods in Stockton, California with $500 per month for 24 months starting in 2019. |
| Alaska Permanent Fund Dividend (APFD)^2^ | The APFD was established in the U.S. state of Alaska in 1976; 25% of revenue from the Trans-Alaska Pipeline System is paid to out annually to Alaska residents. The annual payment has ranged from $331.29 in 1984 to $3,284 in 2022. |
| Rural Income Maintenance Experiment (RIME)^3^ | The Rural Income Maintenance Experiment provided 809 rural, low-income families in the U.S. states of Iowa and North Carolina with negative income tax benefits between 1969-1973. Benefit amounts ranged from 50-100% of participant’s household income. |
| Eastern Band of Cherokees’ casino dividend program^4^ | The casino dividend program began in 1997 in rural North Carolina. The program provides a portion of the casino revenue on the reservation to all Eastern Band of Cherokee tribal members through annual dividends which range from $4,000-6,000 per year. |

**1.** Stockton Economic Empowerment Demonstration website. Available at: <https://www.stocktondemonstration.org/>. Accessed 9/14/2022, 2022.

**2.** Alaska Department of Revenue. *Permanent Fund Dividend.*

**3.** Bawden DL, Harrar WS. Purpose and Design of the Rural Income Maintenance Experiment. *American Journal of Agricultural Economics.* 1977;59(5):855-858.

**4.** Marinescu I. Summary: Universal Basic Income. *Wharton PPI B-School for Public Policy Seminar Summaries.* 2019;11.
