# Supplementary material for: Baltimore community resident and collaborator perspectives on the influence of guaranteed income on health: a formative qualitative study
Source: BMC Public Health. 2024 Sep 18;24:2537. doi: 10.1186/s12889-024-19771-5 (PMC11409528; doi:10.1186/s12889-024-19771-5)
Supplement: Supplementary file 3 — Supplementary Material 3 [file 12889_2024_19771_MOESM3_ESM.pdf]

In-depth Interview Guide for Clients

Study Title: Development of A Framework for Centering Health Equity in Baltimore's Guaranteed Income Pilot
Principal Investigator: Lorraine Dean, ScD
IRB No.: 16992
PI Version Date: Version 2/ 28 June 2021

This guide is focused around three main topic areas: (1) health Indicators that may be affected by income and respondents' thoughts/understanding of the ways that some amount of extra income would affect these indicators and overall health; (2) health outcomes that may be affected by extra income; and (4) community health- how respondents define it; what indicators may be influenced by having more income in a community; and respondents' thoughts on ways that an influx of more income would affect community health.

Beneath each main topic area is an example of a "big" question designed to start the conversation. Beneath each big question is a list of probes that may or may not be used to prompt respondents to continue their conversation. The interviewer may not use every probe in the guide; the probes are there to remind you to consider other aspects of each topic when listening and responding to the respondents.

Please keep in mind that we need to hear from the participants as much as possible. Interviewers must keep their personal opinions and experiences to a minimum.

Thank you for agreeing to answer some questions about how you think extra income could affect your/your clients' health. Your answers will be confidential. Nothing you say will affect your employment or participation in any program. Only in a situation where we feel you are a danger to yourself or others would we intervene to seek help with those properly trained to respond and assist.

Section 1: Individual Background

1. **Please tell me about yourself.**
   Probes: Community, Home/Family**,** School, Children/Partner(s)

Section 2a: Client Experience in Cash-Transfer Programs

[If client is not in cash transfer program, skip to Section 2b.]

1. **Please tell me about the cash transfer program you participated in.**
   Probes:
   - How was the program set up?
   - What was the eligibility criteria for participation?
   - How much money did you receive from the program and with what frequency?
   - What sorts of things did you use the additional income for?
   - How did you prioritize what you spent the money on?

2. **Please share an example of how you used the money provided to you by the program.**

3. **Describe the health impacts, if any, the additional income had on you or your family's health.**
   Probes**:**
   - How much additional income might help your health?
   - Do you think $250 per month would make a difference or not? $500? $1000?

4. **What is your life like now that you have participated in this program?**
   Probes:
   - How is your life similar or different after participation?
   - Community, Home/Family, School/Work/Hobbies, Children/Partner(s)

Section 2b: Non-Cash Transfer Client Experience in Similar Programs

1. **Please describe any services you received from this program.**

2. **Did these services help you save you any money? If you were able to save, what sorts of things did you use the additional money for?**

3. **What difference, if any, did the services from the program have on your life, in general?**
   Probe: How about your health?

4. **What is your life like now that you have participated in this program?**
   Probes:
   - How is your life similar or different after participation?
   - Community, Home/Family, School/Work/Hobbies, Children/Partner(s)

Section 3: Opinions on Guaranteed Income Initiative

1. **How familiar are you with the Baltimore Mayor's new Guaranteed Income initiative?**
   [If not familiar, explain the program as: In February 2021, Mayor Brandon Scott announced his formal commitment to the Mayor's for Guaranteed Income (MGI) Project. Guaranteed income initiatives are place-based initiatives that offer support to all members of an identified area as an unconditional (no work requirements or individual income limits) cash supplement that recipients can use on anything they choose. For example, an ongoing cash assistance program for a period of time (e.g., $500 per month for 12 months) offered to everyone living in an underserved community. So far, the Mayor has appointed a Steering Committee to help identify which communities will be a focus for Baltimore, the amount of assistance, and the length of time eligible residents can receive it.]

2. **What are your thoughts on this type of program?**
   Probes:
   - How do you think it should be set up?
   - *[If in cash transfer program]* In what ways should the initiative be similar or different to the cash transfer programs you've been involved with?
   - How might something like this affect eligibility for other supportive services?

Section 4: Health Effects of Cash Transfers

In this next section, we spend a few minutes discussing how additional income might affect you, your household's health, or the health of your community.

1. **If you or your household were to receive $250-$1000 more income per month, to what extent, if any, would the additional income impact on your health?** *[First ask open-ended and then probe on specific aspects of health]*
   Probes:
   - how you eat?
   - physical activity?
   - diseases like diabetes, high blood pressure, heart disease that you may get from diet, lifestyle, or genes

- diseases like COVID19, sexually transmitted diseases that you may get from other people?
- maternal (pregnancy) health?
- child health?
- mental health?
- substance use?
- exposure to or involvement in violence?
- exposure to environmental hazards like pollution (lead/mold in house, air pollution, water, or noise pollution)?
- taking care of others who may be sick or disabled?
- access to or use of medical care or medications?

2. **How do you think a cash transfer initiative might influence your community if at all?**
   Probe: Your community's health e.g. access to *quality* housing, jobs, education, childcare, transportation, greenspace, strong community ties/social fabric, safe environment/low crime, violence?

Section 5: Sociodemographic information

I want to ask you about some demographic information for description of people who participated in this study again, this section is also completely voluntary, please only share information you feel comfortable sharing.

Age: ______________________________

Educational Attainment: ______________________________

Racial/Ethnic Identities: ______________________________

Sexual Orientation: ______________________________

Gender Presentation: ______________________________

Relationship Status: ______________________________

Current Occupation(s): ______________________________

Disability: ______________________________

Annual income: ______________________________

Source(s) of Income: ______________________________

Number of dependents in household: ______________________________
